# Visualization of gender, race, citizenship and academic performance in association with career outcomes of 15-year biomedical doctoral alumni at a public research university

**DOI:** 10.1371/journal.pone.0197473

**Published:** 2018-05-17

**Authors:** Ambika Mathur, Annmarie Cano, Michael Kohl, Nisansala S. Muthunayake, Prassanna Vaidyanathan, Mary E. Wood, Mustafa Ziyad

**Affiliations:** 1 The Graduate School, Wayne State University, Detroit, Michigan, United States of America; 2 Office of Scientific Training, Workforce Development and Diversity, Wayne State University, Detroit, Michigan, United States of America; 3 Department of Chemistry, Wayne State University, Detroit, Michigan, United States of America; Charles P. Darby Children's Research Institute, UNITED STATES

## Abstract

It has long been thought that biomedical doctoral students pursue careers primarily as tenure-track/tenured faculty at research institutions. Recent reports showed, however, that the majority of biomedical doctoral alumni engage in a variety of careers. Wayne State University (WSU) undertook a project to understand the career trajectories of its biomedical doctoral alumni to create programs to better prepare its students for careers in multiple pathways. Data were collected on career outcomes of WSU’s biomedical doctoral alumni who graduated in a 15-year period from 1999–2014. Careers were classified into three tiers by Employment Sector, Career Types and Job Functions and career paths were examined by alumni gender, race, U.S. citizenship status, and association with certain academic characteristics. Several statistically significant differences in career paths among all demographics were found. For example, women were more likely to be in teaching and providing healthcare, men in faculty and research; Black alumni pursued careers in Government at higher rates and Whites in For-Profit careers; Asians and non-U.S. citizens spent more time in training positions than others. There was no association of academic characteristics such as GRE, GPA, and Time-to-Degree completion with careers in the two largest sectors of Academia or For-profit. Since our trainees are engaged in this rich variety of careers essential to advancing biomedical science and research nationally, it is imperative for the graduate training community to embrace all careers as successful, and transform the model for biomedical doctoral training to foster student success across this broad career spectrum.

## Introduction

Academic institutions have long held the belief that biomedical doctoral students pursue careers primarily as tenure-track/tenured faculty in research institutions, and therefore training programs focused almost exclusively on preparing students for academic careers. Until recently, study section reviewers of training grants from federal agencies such as the National Institutes of Health (NIH) recognized academic careers as the primary outcome of success, further reinforcing this belief. Recent reports regarding career outcomes of doctoral alumni, however, showed that, in reality, almost 75% of all biomedical doctoral students engage in a variety of careers beyond academia, including for-profit, government, and non-profit sectors [1, 2]. It is important to highlight the range of careers in which our doctoral alumni are engaged so that policy makers, taxpayers and Congress, as well as current students, future doctoral students and doctoral program faculty appreciate the impact of our biomedical graduates on biomedical science nationally and globally. Yet, the majority of doctoral programs were not collecting data on career outcomes of their alumni.

With increasing calls for transparency of reporting career outcomes [3–6], some academic institutions posted their outcomes data publicly [7–11]; however, it quickly became apparent that these institutions were using a variety of taxonomies to define the same job sectors, types and functions, making it difficult to aggregate data to report collective career trends nationally. A number of groups, including members of the National Institutes of Health Broadening Experiences in Scientific Training (NIH-BEST) grantee consortium, Association of American Medical Colleges’ Graduate Research Education and Training (AAMC GREAT) group, and Rescuing Biomedical Research (RBR), therefore came together in 2017 and proposed a common three-tier taxonomy to standardize these classifications: Tier 1 includes five Employment Sectors, Tier 2 five Career Types, and Tier 3 26 Job Functions [12], and as shown in S1 Table.

Additionally, very few reports addressed demographics of students engaged in these various careers [13, 14], resulting in assumptions, not necessarily based on evidence, among the graduate training faculty and administration about career choices of women, students from under-represented backgrounds, and non-U.S. citizens. Another widely held perception is that only the students with certain academic characteristics, such as high GRE scores, high GPAs and low Time-to-Degree completion, opt for tenure-/tenure-track faculty positions while others “settle” for “other” careers.

Wayne State University (WSU) is a comprehensive research institution with an enrollment that includes 1,500 doctoral students in 75 doctoral programs. Like other academic institutions, we also operated under the assumption that our doctoral students engage in careers almost exclusively in academia. Our training models were designed to prepare students solely for academic careers. To ensure that we were accurately understanding the short- and long- term career pathways of our Ph.D. recipients, we collected career outcomes data of doctoral alumni who graduated over a 15-year window from 1999 to 2014 [7].

We used the career in which the alumnus was engaged at the point in time of the data collection and “binned” these data in aggregate for alumni based on number of years from graduation (0–5 years; 6–10 years; and 10–15 years post-graduation). These aggregated career outcomes data were then classified per the unified three-tier taxonomy [12], and used to ask the following questions: (a) in which Employment Sectors, Career Types, and Job Functions are WSU’s alumni engaged; (b) is there a distinction between the types of careers pursued based on gender, race and U.S. citizenship status of alumni; and (c) is there a correlation of career outcomes with academic characteristics such as GRE scores, doctoral GPA, and Time-to-PhD Degree completion? To our knowledge, this is the first comprehensive report examining the demographics and academic preparedness of biomedical doctoral alumni as they relate to short and long-term career outcomes.

## Materials and methods

### Alumni census project

In 2015 WSU’s Graduate School launched an Alumni Census Project in which the current employment information of 866/950 (91%) biomedical doctoral alumni who graduated from 1999–2014 from biomedically-related programs were collected, as previously described [7]. These programs are Anatomy and Cell Biology; Biochemistry and Molecular Biology; Biological Sciences; Biomedical Engineering; Cancer Biology/Oncology; Chemistry; Communication Sciences and Disorders; Immunology and Microbiology; Medical Physics; Molecular Genetics and Genomics; Nutrition and Food Sciences; Pathology; Pharmaceutical Sciences; Pharmacology; Physiology; Psychiatry and Behavioral Neurosciences; and Psychology. The information gathered included a direct survey of alumni to indicate their current job placement, as well as information gathered directly from graduate programs and graduate faculty. Alumni were also asked to answer a series of questions about their career trajectories, including information on their first placement, the length of time they have been with their current employer as well as their various job titles over time to provide a rich view of their career progression. The complete survey is available in the S1 File. Self-reported employment data were validated using alumni institutional websites, federal funding agency and publication records, Google, LinkedIn, and other professional social media sites. Only one alumnus was unemployed at this point in time (0.1%); we have omitted this student from further consideration and report outcomes for the remaining 865 alumni.

### Ethical approval

This project was conducted with approval from Wayne State University’s Institutional Review Board on the Use of Human Subjects, IRB#094013B3E.

### Aggregate data reporting

All data are reported in aggregate or with identifiable information removed. Data in which a group is below 4% are not reported in order to maintain confidentiality and anonymity of the individual(s).

### Characteristics of alumni in each tier

The 865 alumni include 459 women (53%), 406 men (47%); 464 White (54%), 334 Asian (39%), 48 Black (5%), 19 total Native American, Pacific Islander race (2%); 478 U.S. citizens/permanent residents (55%), and 387 non-U.S. citizens (45%), as shown in Table 1. We have additionally shown gender, race and citizenship status for each category in Table 1. We compared the career choices of alumni based on gender (men and women); race (Asian, Black, White; however we are not reporting the outcomes of alumni that have small numbers to maintain their confidentiality and because statistical power is too low in these groups); and citizenship status (U.S. citizen/permanent resident or non-U.S. citizen).

**Table 1:** Gender, race, and citizenship status of 15 year biomedical doctoral alumni (n = 865).

	Women	Men	White	Asian	Black	Others	US citizen	Non-US citizen
Total (865)	459	406	464	334	48	19	478	387
White (464)	264	200	464	-	-	-	384	80
Asian (334)	153	181	-	334	-	-	50	284
Black (48)	31	17	-	-	48		33	15
Other (19)	11	8	-	-		19	11	8
US citizen (478)	290	188	384	50	33	11	478	-
Non-US citizen (387)	169	218	80	284	15	8	-	387

### Data reporting and visualization

**Fig 1:**
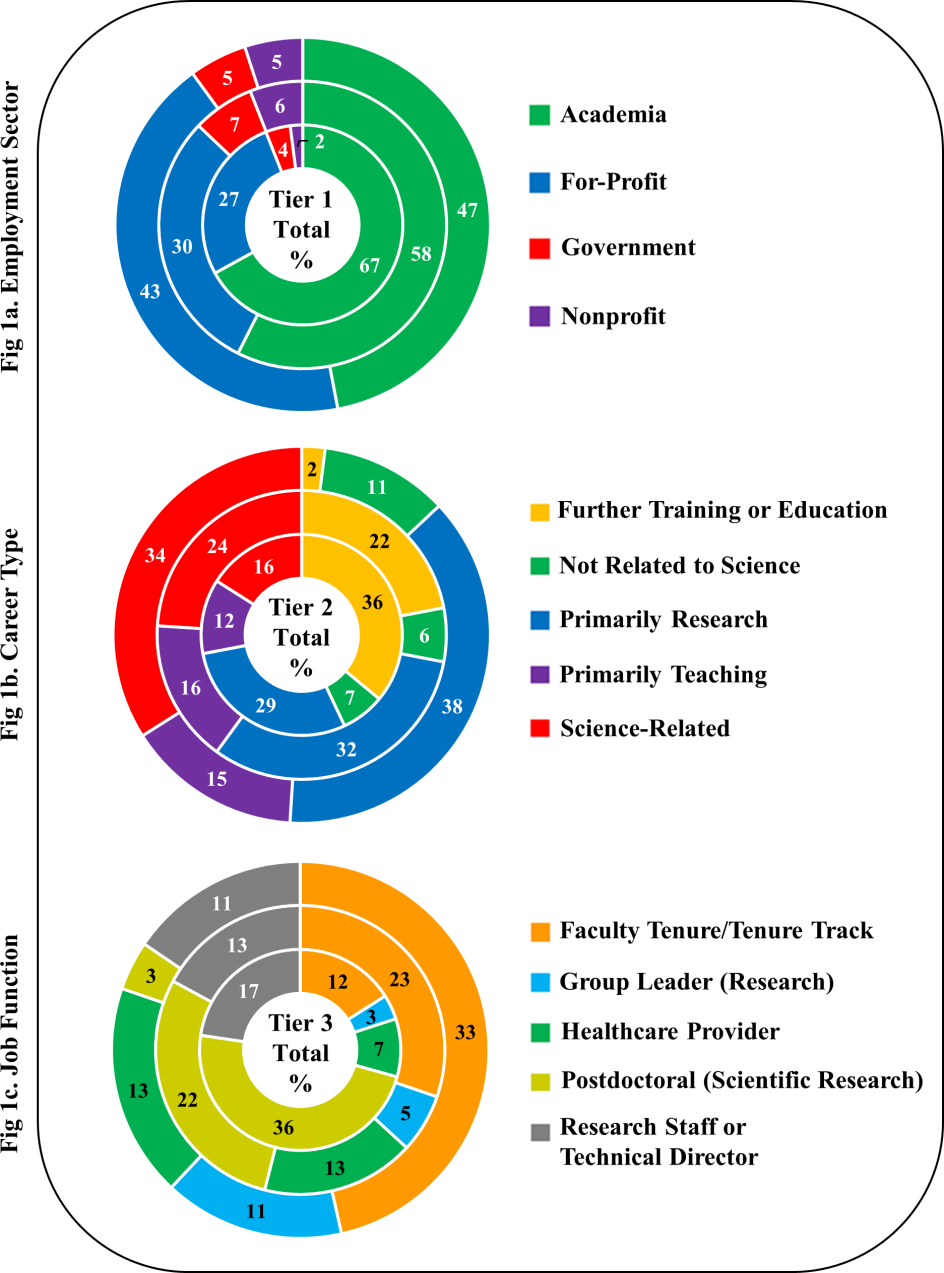
Career outcomes (by %) of WSU’s biomedical doctoral alumni. Fig 1 represents outcomes of alumni in Tier-1 (Employment Sector), Tier-2 (Career Type), and Tier 3 (Job Functions) in the three times windows (0–5 years, inner circle; 6–10 years, middle circle; and 11–15 years, outer circle).

**Fig 2:**
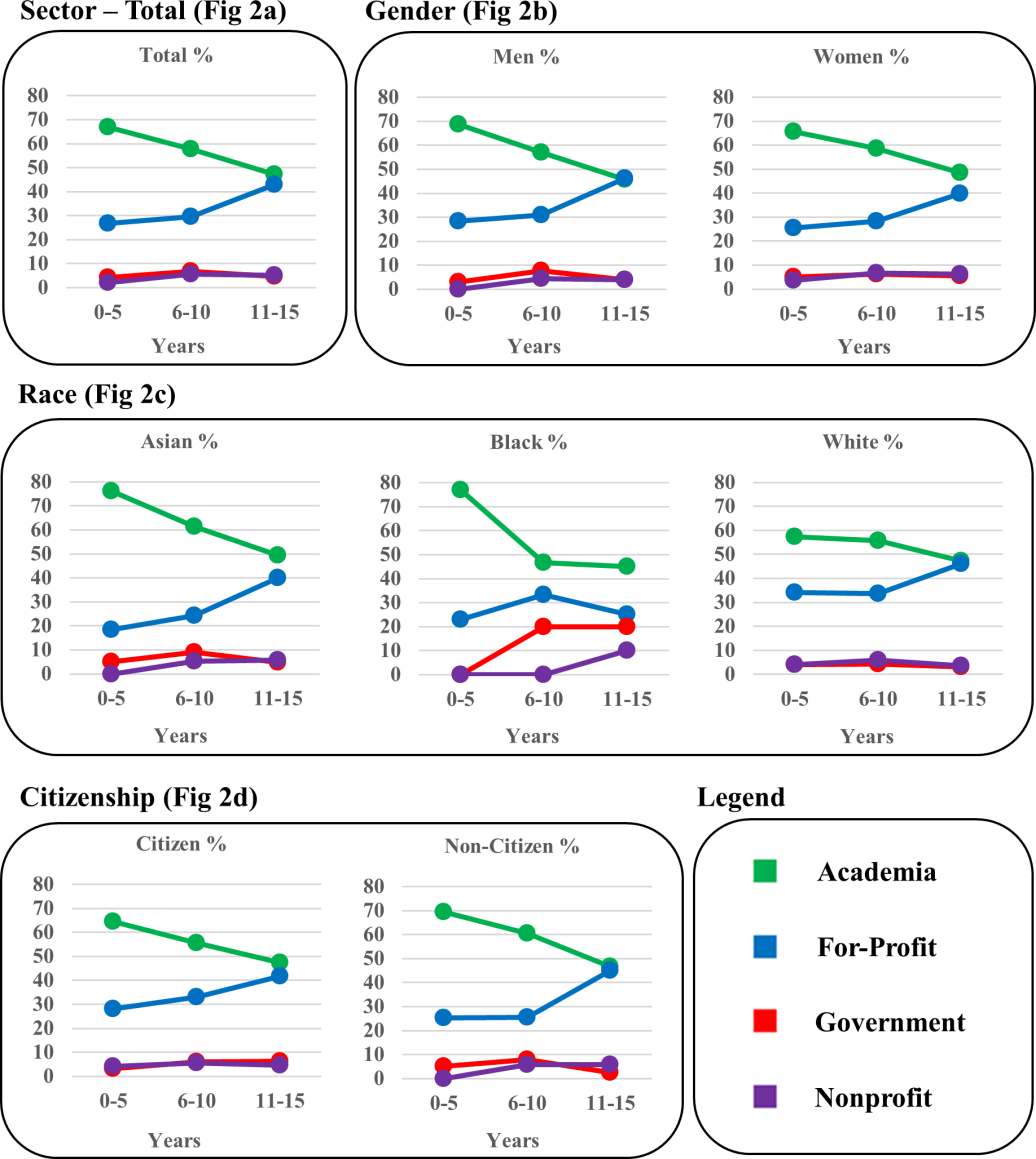
Tier 1 (Employment Sector) outcomes. Employment data are shown as percent of WSU’s biomedical doctoral alumni depicted in three 5-year windows to visualize employment shifts over time following graduation. Fig 2A shows total alumni, Fig 2B shows Gender (men and women), Fig 2C shows Race (Asian, Black, White), and Fig 2D shows U.S. citizenship status (U.S. citizens and non U.S. citizens).

**Fig 3:**
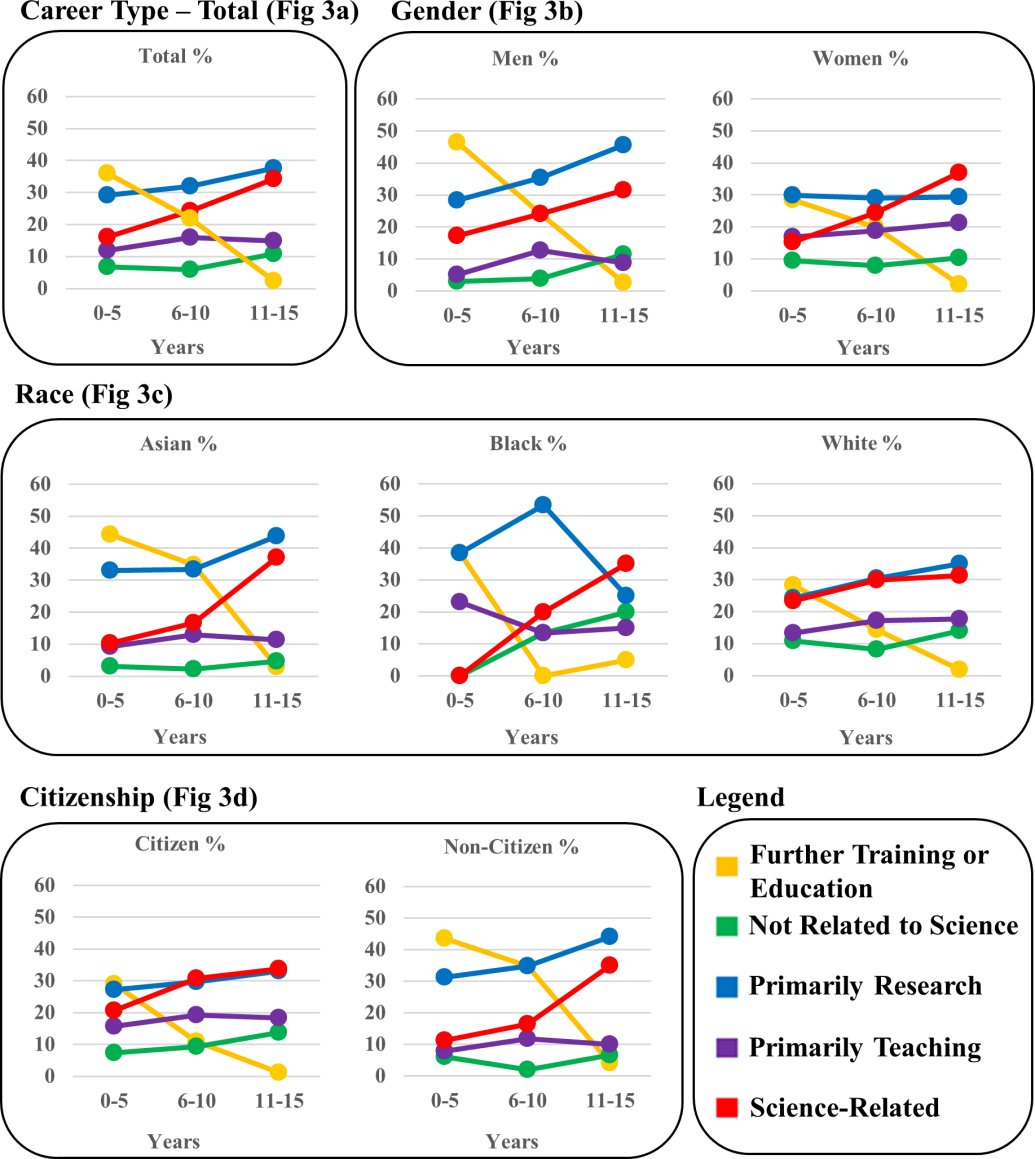
Tier 2 (Career Type) outcomes. Employment data are shown as percent of WSU’s biomedical doctoral alumni depicted in three 5-year windows to visualize employment shifts over time following graduation. Fig 3A shows total alumni, Fig 3B shows Gender (men and women), Fig 3C shows Race (Asian, Black, White), and Fig 3D shows U.S. citizenship status (U.S. citizens and non U.S. citizens).

**Fig 4:**
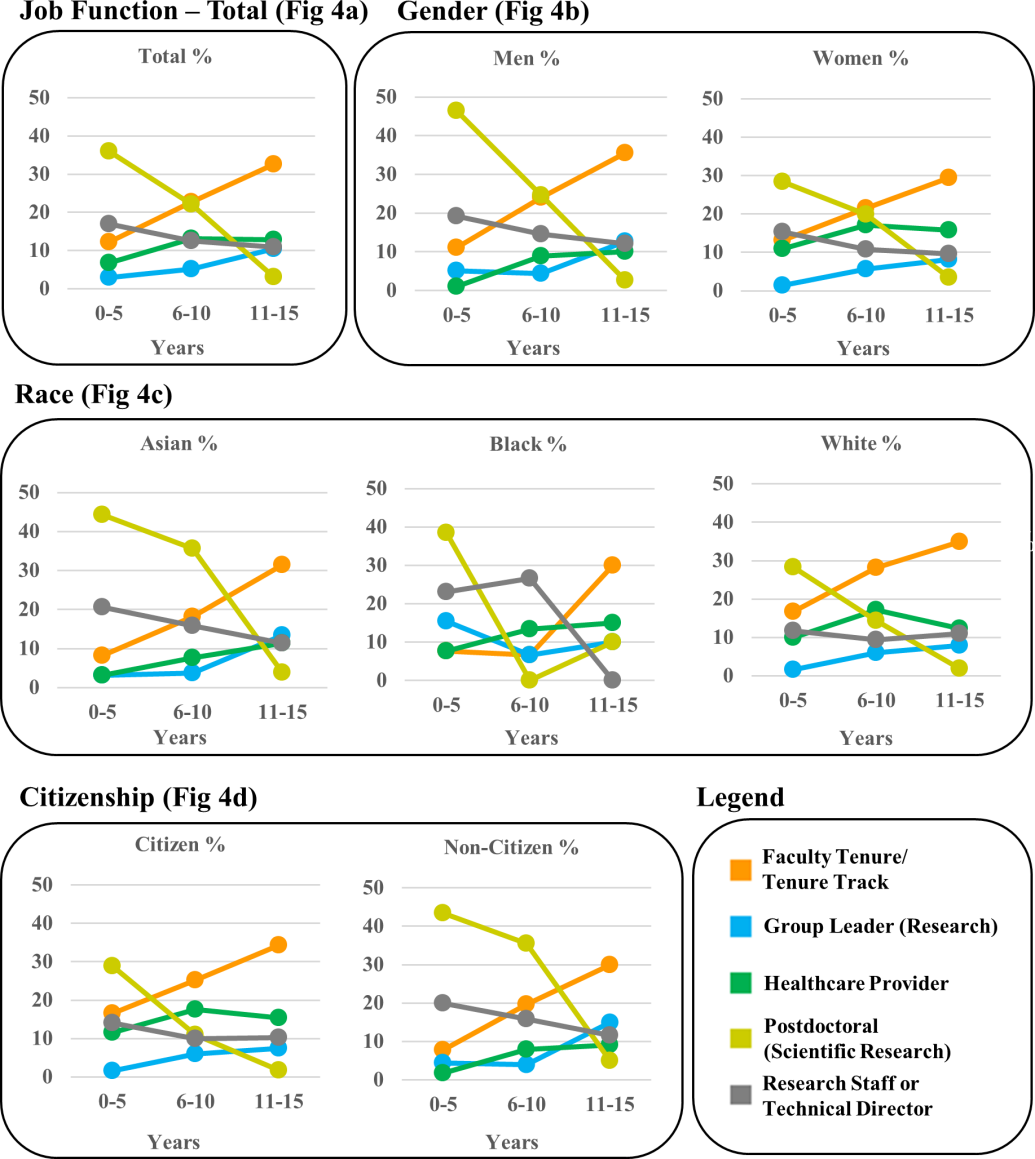
Tier 3 (Job Function) outcomes. Employment data are shown as percent of WSU’s biomedical doctoral alumni depicted in three 5-year windows to visualize employment shifts over time following graduation. Fig 4A shows total alumni, Fig 4B shows Gender (men and women), Fig 4C shows Race (Asian, Black, White), and Fig 4D shows U.S. citizenship status (U.S. citizens and non U.S. citizens).

Career outcomes are reported in percentages; numbers from which these percentages are derived are presented in Tables A-X in S2 Table. Categories with fewer than 4% alumni are not reported in order to preserve confidentiality because in some groups only 1–2 people may be represented. As we examined our data, we realized that the distributions of alumni in Employment Sectors, Career Types and Job Functions change over time. Since most of these changes were seen in 5-year windows, we have depicted all data in these three 5-year windows to visualize employment shifts; *i*.*e*., Window 1 (0–5 years), Window 2 (6–10 years), and Window 3 (11–15 years) immediately following graduation. Note that in this manuscript, the trajectory of each alumnus over a 15-year time period is not reported. Rather, the overall alumni aggregate employment data are shown in each time window from years following graduation within each of the three tiers at the specific time of the survey. So, for example, a student who graduated in 2006 would be represented in the 6–10 year block only. Fig 1 summarizes all three Tiers for these time windows, and Figs 2–4 show each Tier by category. To reiterate, these are aggregate data of all alumni in the time window and not movement of individual alumni over time.

### Statistical analysis

Outcome analyses were performed using SPSS version 25 (IBM 2018). Chi-Square (Χ^2^) analyses with follow-up z tests employing a Bonferroni correction were used to test for significantly different proportions of alumni in different Employment Sectors, Career Types, and Job Functions over time. Since time windows contained different sets of participants, between-subjects analyses were conducted. Multinomial logistic regression analyses were then conducted to test for significant interactions between time windows and demographic characteristics. However, none of these interactions were statistically significant and in some cases (example, Race), singularities in the Hessian matrix due to small sample sizes in some cells prevented valid multinomial analysis. In the absence of significant interaction effects and because of the Hessian matrix violations, Chi-Square (Χ^2^) analyses were used to test for significantly different proportions of demographic groups within each tier. As with time windows, post hoc z tests with Bonferroni corrections were used to test for significant effects if the omnibus Chi-square test was significant. Differences among comparison groups were considered to be statistically significant at p < .05. Statistical output tables are reported in Tables A-L in S3 Table. In addition, we conducted multinomial logistic regression analyses to test for significant interactions between combinations of demographic characteristics (e.g., gender, race, and citizenship). However, singularities in the Hessian matrix due to small sample sizes in some cells prevented valid multinomial analysis. Chi-Square (Χ^2^) analyses were also attempted to examine patterns of career outcomes in isolated subsets of alumni; however, small and n = 0 cell sizes for some categories resulted in uninterpretable results. Because the patterns of findings appear to be similar for these small groups, we decided to present analyses on each demographic variable (rather than combinations of variables) to yield robust results that could be used as a basis for future investigations. Thus, the analyses presented here focus on patterns of career outcomes within each demographic group for the entire 15-year window.

### Association of academic characteristics and career sector outcomes

Academic characteristics assessed were (a) GRE-Quantitative and GRE-Verbal scores; (b) cumulative GPA at doctoral graduation; and (c) time to doctoral degree completion. Average Time-to-Degree at WSU for biomedical doctoral students is 5.5 years. Logistic regression analyses were used to test whether academic characteristics were associated with Employment Sector outcomes. Significance was determined with a p value < .05.

## Results

### Classification of 15-year career outcomes of WSU’s biomedical doctoral alumni in the three tiers

In our overall alumni outcomes, not all employment sectors, career types and job functions were equally represented (Tables A-X in S2 Table). For Tier-1 (Employment Sector), the careers in which are alumni are employed, in ranked order of frequency, are Academia, For-Profit, Government, and Nonprofit sectors. For Tier-2 (Career Type) alumni engage in careers that are Primarily Research, Science Related, Further Training or Education, and Primarily Teaching, with a small percent in careers Not Related to Science. Careers not related to science include alumni who are working in service industry jobs that have no relevance to science. For Tier 3 (Job Functions) most alumni are employed in the five primary jobs of Faculty-Tenure/Tenure Track (hereafter referred to just as Faculty), followed by Postdoctoral Training, Research Staff/Technical Director, Healthcare Provider, and Group Leader in Research, with all other job functions being 4% or fewer. Job Functions which have fewer than 4% alumni include: Adjunct Teaching; Administration; Business Development, Consulting, and Strategic Alliances; Clinical Research Management; Clinical Services; Data Science, Analytics, and Software Engineering; Entrepreneurship; Function that is Not Directly Related to Science; Intellectual Property and Law; Lecturer/Instructor; Regulatory Affairs; Lecturers; Sales and Marketing; Science Education and Outreach; and Science Policy and Government Affairs. Rather than showing all careers, to keep changes in careers meaningful, we are showing only those careers in which 4% or greater alumni are employed.

#### Total alumni data (Fig 2A)

Almost 90% of alumni are engaged in either Academia or For-profit sectors. The pattern of Employment Sector changed over time, Χ^2^ (6, N = 865) = 20.30, p = .002. Post hoc z tests were performed to determine when alumni representation increased or decreased in particular sectors. While the majority of alumni start out in Academia, more alumni are represented in sectors outside of Academia, especially when comparing Window 1 to Window 3, p < .05. One reason for this drop is that the Academic sector includes postdoctoral and other trainees (19%), and as expected, they move from traineeships to other sectors. The only other significant change is the increase over time in the number of alumni who were employed in the For-Profit sector (p < .05). Very small percentages of alumni pursue careers in Government and Non-profit sectors and no statistically significant changes in alumni representation occur for these sectors.

#### By gender (Fig 2B)

There are no significant differences between men and women in any Employment Sectors, with the trends being the same as those in the total population.

#### By race (Fig 2C)

There was a significant effect of Race on Sector Χ^2^ (6, N = 846) = 20.17, p = .003. A higher proportion of Asians entered Academia compared to Whites (p < .05) whereas a higher proportion of Whites compared to Asians chose careers in For-profit (p < .05). The only other significant difference is seen with Blacks in that they are employed in significantly higher percentages in the Government sector as compared with Asians or Whites (p < .05).

#### By U.S. citizenship status (Fig 2D)

There were no significant differences between U.S. citizen and non-U.S. citizen alumni showed similar patterns in Employment Sectors, with a small increase in the For-profit sector for non-U.S. citizens from Window 1 to Window 3.

#### Total alumni data (Fig 3A)

The majority of alumni are in Primarily Research and Science Related Career Types, with a smaller percentage in Primarily Teaching. A small percentage of alumni is in careers Not Related to Science. The distribution of Career Types changed over time, Χ^2^ (8, N = 865) = 116.05, p = .0001. As expected and desired, there was a significant decline in the percentage of alumni in Further Training after Window 1 (Window 1 to 2 p < .05, Window 2 to 3 p < .05). There is a significant increase in the number of alumni in Science Related careers between Windows 1 and 2 (p < .05) and an increase in the number of alumni in careers Not Related to Science from Window 1 to 3 (p < .05).

#### By gender (Fig 3B)

Men and women differed in their Career Types, Χ^2^ (3, N = 865) = 22.72, p = .0001. Significantly more women were in Primarily Teaching careers (p < .05), and more men in Primarily Research careers (p < .05).

#### By race (Fig 3C)

Racial group distributions, Χ^2^ (8, N = 846) = 47.27, p = .0001, were accounted for by Asians pursuing Further Training in higher proportions compared to White and Black alumni (p < .05), and Whites choosing Career Types that were Not Related to Science at a higher rate than Asians and Blacks (p < .05).

#### By U.S. citizenship status (Fig 3D)

Career Type was associated with citizenship status, Χ^2^ (4, N = 865) = 55.94, p = .0001. U.S. citizens were more likely to be in Primarily Teaching, Not Science-Related, or Science-related careers (p < .05 for all comparisons). Significantly more non-U.S. citizens were employed in Further Training and Primarily Research careers (p < .05 for both).

#### Total alumni data (Fig 4A)

There were significant changes in the proportions of alumni in Job Functions over the time windows, Χ^2^ (8, N = 636) = 120.72, p = .0001. As expected, the proportion of alumni who start in Postdoctoral or related traineeships immediately following graduation is followed by declines over the time windows (p < .05). More alumni are represented in Faculty and Technical Director or Group Leader positions after Window 1 (p < .05).

#### By gender (Fig 4B)

The significant Chi-square for gender, Χ^2^ (4, N = 636) = 16.36, p = .003 was accounted for by the fact that women choose careers as Healthcare Providers at a significantly higher rate than men (p < .05).

#### By race (Fig 4C)

Significant racial group differences in Job Functions also emerged, Χ^2^ (8, N = 621) = 39.26, p = .0001, with differences between Asians and Whites seeming to account for this overall effect. Asians were more likely to be Postdoctoral (Scientific Research) scholars compared to Whites (p < .05) whereas White alumni were significantly more likely to be Faculty and Healthcare providers (p < .05 for both comparisons).

#### By U.S. citizenship status (Fig 4D)

Significant citizenship effects, Χ^2^ (4, N = 636) = 51.40, p = .0001, were due to a significantly higher percentage of non-U.S. citizens entering Postdoctoral (Scientific Training) (p < .05), as well as higher percentages of U.S. citizens pursuing careers as Health Care Providers and Faculty (p < .05 for both comparisons).

### Academic characteristics and career sector in Academia and For-profit sectors

We examined the association of academic characteristics with the two largest Employment Sectors of Academia and For-profit, since participation in other sectors is too small to allow for meaningful comparisons. Please note that not all students take the GRE since it is not a requirement for admission to all biomedical programs at WSU and therefore the numbers do not total 865. Data are expressed as % alumni in each score/year range.

**Fig 5:**
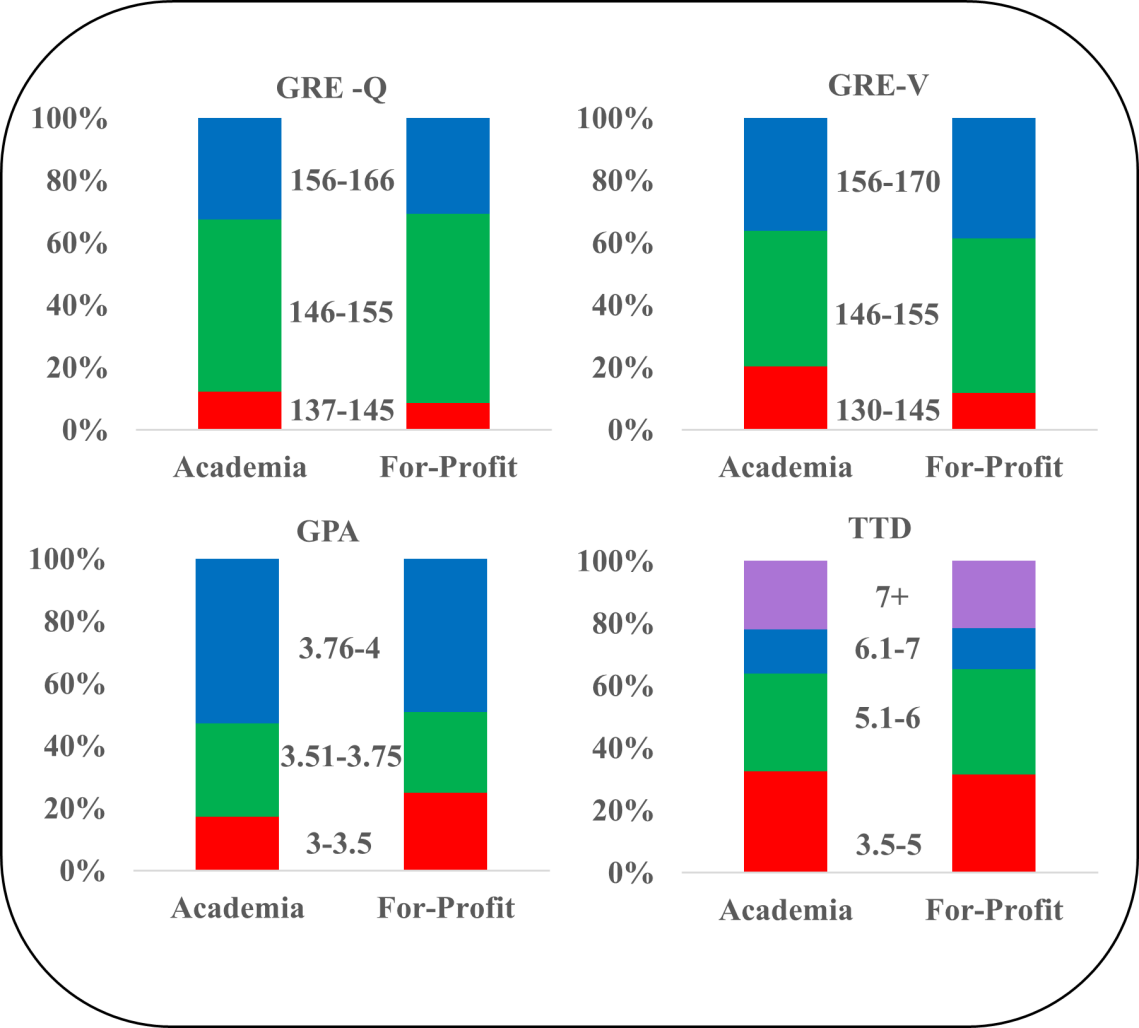
Academic characteristics of WSU’s biomedical doctoral alumni. GRE Quantitative scores (GRE-Q) is shown in blocks of scores of 137–145, 146–145, 146–155; GRE-Verbal (GRE-V) is shown in blocks of scores of 130–145, 146–155, 156–170; cumulative GPA at time of doctoral graduation as blocks of 3.0–3.5, 3.51–3.75, 3.76–4.0; and Time-to-Degree completion (TTD) in blocks of 3.5–5.0, 5.1–6.0, 6.1–7, 7+ years. GRE scores are shown for the 348 alumni who took the GRE exam and are employed in the two sectors of Academia and For-Profit; GPA and TTD are shown for the 779 alumni employed in these two sectors of Academia and For-Profit.

There are no statistically significant differences in any of the academic characteristics examined between alumni in Academia and For-profit sectors. Fig 5 shows an equivalent spread of GRE-Quantitative scores, GRE-Verbal scores, cumulative GPA, and Time-to-Degree completion of alumni between these sectors.

## Discussion

In response to national calls for transparency [1–3, 15], WSU tracked and classified the career outcomes of 91% (865/950) biomedical doctoral alumni who graduated from 1999–2014 [7]. In this report we share our outcomes and show how patterns of alumni careers change over time as alumni apparently move from postdoctoral training to long-term careers in various areas. We believe that this is the first report to comprehensively examine the relationship between demographics of alumni and associations of their academic characteristics with short and long-term career outcomes.

About 92% of our biomedical doctoral alumni are employed in careers related to research and science. This finding counters current literature that argues that the PhD pipeline is producing too many graduates for not only academia, but also for industry and government [2, 16]. While we did not survey our alumni for the reasons behind their career choices, satisfaction surveys indicate that the majority of alumni were very satisfied with their eventual career decisions across all sectors (data not shown).

Overall we find that our alumni do not necessarily remain in a single Employment Sector. We do not know if this change of employment areas is purposeful or is influenced by other decisions such as financial, family, and need to remain in certain geographical locations, among others. Regardless, it is clear that for whatever reason, alumni distribution in careers changes over time, emphasizing the mobility [17] and the “transferrable skills” that doctoral training provides to ensure success in diverse jobs as they move from the Academic sector (mainly as Postdoctoral trainees) to jobs mainly in the For-Profit sector. For overall Career Types, as expected, alumni “graduate” from Further Training and show significant increases in Science Related, but also to a smaller extent in Not Related to Science career types. A review of these careers show that our alumni are engaged in interesting and successful careers even though these are not in science. We find that more alumni engage over time in Tenure-track/Tenure Faculty, Research Staff or Technical Director, and Group Leader (Research) Job Functions overall. The percent of our alumni in Faculty positions surprised us since there are many publications about the loss of students from these job functions indicating that students lose interest in academic careers during their doctoral training for a variety of reasons [18] including finding academic careers “unattractive”, intense competition, stress, lack of research dollars, low pay and long training periods, as well as personal values [15, 17, 19, 20]. We do not yet understand this difference with our alumni and plan to conduct detailed surveys in the future to address the reasons for this outcome.

Gender does not correlate with differences in Employment Sectors, but men and women show interesting differences in Career Types and Job Functions. More women engage in Primarily Teaching careers and as Healthcare Providers, while more men engage in Primarily Research careers. While we do not know the reasons for these differences, recent discussion in the literature implies that women prefer jobs providing more flexibility such as teaching and health care over jobs in academia because of family obligations and work-life balance [21–24].

Race is associated significantly with many outcomes among our alumni in all three tiers. For the Employment Sector, the largest differences observed are that Asian alumni enter Academia at higher rates compared with Whites and Blacks; Black alumni are more likely to be in the Government sector compared with Asians and Whites; and White alumni are more likely than Asians to engage in For-profit sectors. While no race-based differences are found among Primarily Research, Primarily Teaching or Science Related Career Types, more Asians tend to remain in Further Training compared with Blacks and Whites, and White alumni choose Not Related to Science careers at a higher rate than Asians or Blacks. For Job Functions, most significant differences are seen among Asian and White alumni. Our White alumni pursue careers as faculty and Healthcare Providers at higher rates than Asians, and Asians remain for longer durations in Postdoctoral Training compared with Whites. Race and ethnicity patterns among biomedical doctoral alumni have been described in several studies [25–29], and well-examined by Gibbs et al [13] who found that Ph.D. recipients from under-represented backgrounds chose job functions other than faculty. Our Black alumni reflect this national trend and diverge from their White and Asian counterparts by moving from Postdoctoral training to Government and Non-profit sectors. In future studies, we will survey our alumni to better understand the reasons for their career choices.

There is a dearth of literature on the career outcomes of U.S. citizens and non-U.S. citizens. A recent study shows that significantly more U.S. postdoctoral alumni pursue careers in for-profit ventures and obtained more tenure/tenure-track positions than international postdocs [4]. At WSU, U.S. citizens and non-citizen alumni are engaged at similar rates in all Employment Sectors. Divergence occurs at the Career Type tier where more U.S. citizens engage in Primarily Teaching, Science Related and Not Related to Science careers, and conversely, more non-U.S. citizens engage in Primarily Research and remain longer in Further Training than citizens. A greater percentage of U.S. citizens pursue jobs as Faculty and Healthcare Providers, and more non-citizens, unfortunately, remain in postdoctoral positions, possibly due to issues related to visa status.

Regardless of gender, race, and U.S. citizenship status, our data show that alumni engage in a variety of careers. Academic institutions, faculty, and funding agencies should respect these multiple career pathways because scientists and researchers in these areas contribute to and enhance scientific discovery in new and meaningful ways. Our alumni shape public policy in government jobs, lead research and innovation efforts in industry, influence public perception of science through communications, and teach the next generation of science researchers and practitioners. We must embrace these careers as successful outcomes of doctoral training and make it acceptable for students to explore and identify their career interests in programs developed by the graduate training community, such as the NIH-funded Broadening Experiences in Scientific Training (BEST) programs [30] including WSU’s BEST program [31] that are designed specifically for this purpose. In these programs, students are provided exposure to diverse careers and receive structured training in transferrable skills that transcend and ensure success across all careers [32, 33].

One of the most important findings from our study is that alumni in the two largest employment sectors of Academia and For-profit show very similar academic characteristics. There is no statistically significant difference between the two groups in either GRE scores, cumulative GPA, or Time-to-Degree completion. This debunks the myth, for our institution at least, that students who have “better” academic characteristics engage in faculty careers while “other” students settle for careers in other sectors. These data should empower students entering doctoral programs to have honest discussions about their career aspirations with their research advisors and to explore careers outside the professoriate, if they so choose.

In summary, WSU’s 15-year doctoral biomedical alumni are engaged in a variety of careers. Differences are seen among careers of alumni by gender, race, and U.S. citizenship status. There is no correlation of academic characteristics examined of alumni with careers in Academia and For-profit sectors. In addition to disciplinary research training, it is imperative for the graduate training community to embrace all science and research-related careers as successful, and transform training models such that biomedical doctoral students are provided with the appropriate professional development skills to succeed in a broad spectrum of careers. Modernizing curricula and providing opportunities for experiential learning and engaging the private sector is also required to better align education with career paths and empower students to make informed career decisions [18, 19, 32]. BEST consortium members are beginning to use this common three-tier taxonomy to uniformly aggregate career outcomes data at the national level [34]. We hope that other institutions will also join this effort to develop a single robust national collective dataset that will help demonstrate the impact of doctoral training outcomes to students, faculty and society as a whole, and transform biomedical doctoral training models.

## Supporting information

S1 Table Three-tier taxonomy categories by Tier 1-Employment Sector, Tier 2-Career Type, and Tier 3-Job function. (DOCX)

S2 Table Data sets from WSU alumni census project, N = 865 biomedical doctoral recipients used to create the data shown in Figs 1–4. Tables A-H in S2 Table for Employment Sector. Table A: Total alumni data for Figs 1A and 2A; Table B: By gender (men) for Fig 2B; Table C: By gender (women) for Fig 2B; Table D: by race (Asian) for Fig 2C; Table E: by race (Black) for Fig 2C; Table F: by race (White) for Fig 2C; Table G: by U.S. citizenship status (U.S. citizens) for Fig 2D; and Table H: by U.S. citizenship status (non-U.S. citizens) for Fig 2D. Tables I-P in S2 Table for Career Type. Table I: Total alumni data for Figs 1B and 3A; Table J: By gender (men) for Fig 3B; Table K: By gender (women) for Fig 3B; Table L: by race (Asian) for Fig 2C; Table M: by race (Black) for Fig 3C; Table N: by race (White) for Fig 3C; Table O: by U.S. citizenship status (U.S. citizens) for Fig 3D; and Table P: by U.S. citizenship status (non-U.S. citizens) for Fig 3D. Tables Q-P in S2 Table for Job Function. Table Q: Total alumni data for Figs 1C and 4A; Table R: By gender (men) for Fig 4B; Table S: By gender (women) for Fig 4B; Table T: by race (Asian) for Fig 4C; Table U: by race (Black) for Fig 4C; Table V: by race (White) for Fig 4C; Table W: by U.S. citizenship status (U.S. citizens) for Fig 4D; and Table X: by U.S. citizenship status (non-U.S. citizens) for Fig 4D.
(DOCX)

S3 Table Statistical analyses of data shown in Figs 1–5. Analyses are shown in Tables A-D for Employment Sector (Table A for total; Table B by gender; Table C by race, and Table D by U.S. citizenship status); Tables E-H for Career Type (Table E for total; Table F by gender; Table G by race, and Table H by U.S. citizenship status); and Tables I-L for Job Function (Table I for total; Table J by gender; Table K by race, and Table L by U.S. citizenship status). Neither GRE-Q nor GRE-V significantly predicted the likelihood of entering the Academic or For-Profit Employment Sector (GRE-Q B = -.004, SE = .018, Wald = .06, p = .81; GRE-V, B = -.016, SE = .016, Wald = 1.02, p = .31. Neither GPA nor TTD significantly predicted the likelihood of employment the Academic or For-Profit Employment Sector, (GPA B = -.016, SE = .342, Wald = .032, p = .86; TTD B = -.027, SE = .041, Wald = .444, p = .51).
(DOCX)

S1 File Survey instrument for data collected in alumni census project. (PDF)
