# Supplementary material for: Visualization of gender, race, citizenship and academic performance in association with career outcomes of 15-year biomedical doctoral alumni at a public research university
Source: PLoS One. 2018 May 17;13(5):e0197473. doi: 10.1371/journal.pone.0197473 (PMC5957427; doi:10.1371/journal.pone.0197473)
Supplement: S1 Table — (DOCX) [file pone.0197473.s001.docx]

| **Tier 1: Employment Sectors** | **Tier 2: Career Types** | **Tier 3: Job Functions** |
| --- | --- | --- |
| Academia | Primarily Research | Administration |
| Government | Primarily Teaching | Business Development, Consulting, and Strategic Alliances |
| For-Profit | Science-related | Clinical Research Management |
| Nonprofit | Not-related to science | Clinical Services |
| Other | Further training or education | Data Science, Analytics, and Software Engineering |
|  |  | Entrepreneurship |
|  |  | Faculty: non-tenure track |
|  |  | Faculty: tenured/tenure track |
|  |  | Faculty: track unclear or not applicable |
|  |  | Full-time Teaching Staff/Instructor |
|  |  | Group Leader (research) |
|  |  | Healthcare Provider |
|  |  | Intellectual Property and Law |
|  |  | Part-time Teaching Staff/Adjunct |
|  |  | Postdoctoral Research |
|  |  | Regulatory Affairs |
|  |  | Research Staff or Technical Director |
|  |  | Sales and Marketing |
|  |  | Science Education and Outreach |
|  |  | Science Policy and Government Affairs |
|  |  | Science Writing and Communication |
|  |  | Technical Support and Product Development |
|  |  | Completing further education or training |
|  |  | Other |
|  |  | Deceased/retired |
