# Supplementary material for: Visualization of gender, race, citizenship and academic performance in association with career outcomes of 15-year biomedical doctoral alumni at a public research university
Source: PLoS One. 2018 May 17;13(5):e0197473. doi: 10.1371/journal.pone.0197473 (PMC5957427; doi:10.1371/journal.pone.0197473)
Supplement: S2 Table — Tables A-H in S2 Table for Employment Sector. Table A: Total alumni data for Figs 1A and 2A; Table B: By gender (men) for Fig 2B; Table C: By gender (women) for Fig 2B; Table D: by race (Asian) for Fig 2C; Table E: by race (Black) for Fig 2C; Table F: by race (White) for Fig 2C; Table G: by U.S. citizenship status (U.S. citizens) for Fig 2D; and Table H: by U.S. citizenship status (non-U.S. citizens) for Fig 2D. Tables I-P in S2 Table for Career Type. Table I: Total alumni data for Figs 1B and 3A; Table J: By gender (men) for Fig 3B; Table K: By gender (women) for Fig 3B; Table L: by race (Asian) for Fig 2C; Table M: by race (Black) for Fig 3C; Table N: by race (White) for Fig 3C; Table O: by U.S. citizenship status (U.S. citizens) for Fig 3D; and Table P: by U.S. citizenship status (non-U.S. citizens) for Fig 3D. Tables Q-P in S2 Table for Job Function. Table Q: Total alumni data for Figs 1C and 4A; Table R: By gender (men) for Fig 4B; Table S: By gender (women) for Fig 4B; Table T: by race (Asian) for Fig 4C; Table U: by race (Black) for Fig 4C; Table V: by race (White) for Fig 4C; Table W: by U.S. citizenship status (U.S. citizens) for Fig 4D; and Table X: by U.S. citizenship status (non-U.S. citizens) for Fig 4D. (DOCX) [file pone.0197473.s002.docx]

**S2 Table**

**Table A in S2 Table**

| Years | Academia | | For-profit | | Government | | Non-profit | |
| --- | --- | --- | --- | --- | --- | --- | --- | --- |
|  | % | N | % | N | % | N | % | N |
| 0-5 | 67% | 158 | 25% | 63 | 4% | 10 | 2% | 5 |
| 6-10 | 58% | 193 | 28% | 99 | 7% | 23 | 5% | 19 |
| 11-15 | 47% | 139 | 37% | 127 | 4% | 14 | 4% | 15 |

**Table B in S2 Table**

| Years | Academia | | For-profit | | Government | | Non-profit | |
| --- | --- | --- | --- | --- | --- | --- | --- | --- |
|  | % | N | % | N | % | N | % | N |
| 0-5 | 67% | 158 | 25% | 63 | 4% | 10 | 2% | 5 |
| 6-10 | 58% | 193 | 28% | 99 | 7% | 23 | 5% | 19 |
| 11-15 | 47% | 139 | 37% | 127 | 4% | 14 | 4% | 15 |

| Years | Academia | | For-profit | | Government | | Non-profit | |
| --- | --- | --- | --- | --- | --- | --- | --- | --- |
|  | % | N | % | N | % | N | % | N |
| 0-5 | 69% | 68 | 28% | 28 | 3% | 3 | 0% | 0 |
| 6-10 | 57% | 90 | 31% | 49 | 8% | 12 | 4% | 7 |
| 11-15 | 46% | 68 | 46% | 69 | 4% | 6 | 4% | 6 |

**Table C in S2 Table**

| Years | Academia | | For-profit | | Government | | Non-profit | |
| --- | --- | --- | --- | --- | --- | --- | --- | --- |
|  | % | N | % | N | % | N | % | N |
| 0-5 | 66% | 90 | 26% | 35 | 5% | 7 | 4% | 5 |
| 6-10 | 59% | 103 | 28% | 50 | 6% | 11 | 7% | 12 |
| 11-15 | 49% | 71 | 40% | 58 | 5% | 8 | 6% | 9 |

**Table D in S2 Table**

| Years | Academia | | For-profit | | Government | | Non-profit | |
| --- | --- | --- | --- | --- | --- | --- | --- | --- |
|  | % | N | % | N | % | N | % | N |
| 0-5 | 76% | 74 | 19% | 18 | 5% | 5 | 0% | 0 |
| 6-10 | 61% | 81 | 24% | 32 | 9% | 12 | 5% | 7 |
| 11-15 | 50% | 52 | 40% | 42 | 5% | 5 | 6% | 6 |

**Table E in S2 Table**

| Years | Academia | | For-profit | | Government | | Non-profit | |
| --- | --- | --- | --- | --- | --- | --- | --- | --- |
|  | % | N | % | N | % | N | % | N |
| 0-5 | 77% | 10 | 23% | 3 | 0% | 0 | 0% | 0 |
| 6-10 | 47% | 7 | 33% | 5 | 20% | 3 | 0% | 0 |
| 11-15 | 45% | 9 | 25% | 5 | 20% | 4 | 10% | 2 |

| Years | Academia | | For-profit | | Government | | Non-profit | |
| --- | --- | --- | --- | --- | --- | --- | --- | --- |
|  | % | V | % | N | % | N | % | N |
| 0-5 | 70% | 80 | 25% | 29 | 5% | 6 | 0% | 0 |
| 6-10 | 61% | 92 | 26% | 39 | 8% | 12 | 6% | 9 |
| 11-15 | 47% | 56 | 45% | 54 | 3% | 3 | 6% | 7 |

**Table F in S2 Table**

**Table G in S2 Table**

| Years | Academia | | For-profit | | Government | | Non-profit | |
| --- | --- | --- | --- | --- | --- | --- | --- | --- |
|  | % | N | % | N | % | N | % | N |
| 0-5 | 58% | 69 | 34% | 41 | 4% | 5 | 4% | 5 |
| 6-10 | 56% | 101 | 34% | 61 | 4% | 8 | 6% | 11 |
| 11-15 | 47% | 77 | 46% | 75 | 3% | 5 | 4% | 6 |

**Table H in S2 Table**

| Years | Academia | | For-profit | | Government | | Non-profit | |
| --- | --- | --- | --- | --- | --- | --- | --- | --- |
|  | % | N | % | N | % | N | % | N |
| 0-5 | 64% | 78 | 28% | 34 | 3% | 4 | 4% | 5 |
| 6-10 | 55% | 101 | 33% | 60 | 6% | 11 | 5% | 10 |
| 11-15 | 47% | 83 | 42% | 73 | 6% | 11 | 5% | 8 |

**Table I in S2 Table**

| Years | Further Training | | Not Related to Science | | Primarily Research | | Primarily Teaching | | Science Related | |
| --- | --- | --- | --- | --- | --- | --- | --- | --- | --- | --- |
|  | % | N | % | N | % | N | % | N | % | N |
| 0-5 | 36% | 85 | 7% | 16 | 29% | 69 | 12% | 28 | 16% | 38 |
| 6-10 | 22% | 73 | 6% | 20 | 32% | 107 | 16% | 53 | 24% | 81 |
| 11-15 | 2% | 7 | 11% | 32 | 38% | 111 | 15% | 44 | 34% | 101 |

**Table J in S2 Table**

| Years | Further Training | | Not Related to Science | | Primarily Research | | Primarily Teaching | | Science Related | |
| --- | --- | --- | --- | --- | --- | --- | --- | --- | --- | --- |
|  | % | N | % | N | % | N | % | N | % | N |
| 0-5 | 46% | 46 | 3% | 3 | 28% | 28 | 5% | 5 | 17% | 17 |
| 6-10 | 24% | 38 | 4% | 6 | 35% | 56 | 13% | 20 | 24% | 38 |
| 11-15 | 3% | 4 | 11% | 17 | 46% | 68 | 9% | 13 | 32% | 47 |

**Table K in S2 Table**

| Years | Further Training | | Not Related to Science | | Primarily Research | | Primarily Teaching | | Science Related | |
| --- | --- | --- | --- | --- | --- | --- | --- | --- | --- | --- |
|  | % | N | % | N | % | N | % | N | % | N |
| 0-5 | 28% | 39 | 9% | 13 | 30% | 41 | 17% | 23 | 15% | 21 |
| 6-10 | 20% | 35 | 8% | 14 | 29% | 51 | 19% | 33 | 24% | 43 |
| 11-15 | 2% | 3 | 10% | 15 | 29% | 43 | 21% | 31 | 37% | 54 |

**Table L in S2 Table**

| Years | Further Training | | Not Related to Science | | Primarily Research | | Primarily Teaching | | Science Related | |
| --- | --- | --- | --- | --- | --- | --- | --- | --- | --- | --- |
|  | % | N | % | N | % | N | % | N | % | N |
| 0-5 | 44% | 43 | 3% | 3 | 33% | 32 | 9% | 9 | 10% | 10 |
| 6-10 | 35% | 46 | 2% | 3 | 33% | 44 | 13% | 17 | 17% | 22 |
| 11-15 | 3% | 3 | 5% | 5 | 44% | 46 | 11% | 12 | 37% | 39 |

| Years | Further Training | | Not Related to Science | | Primarily Research | | Primarily Teaching | | Science Related | |
| --- | --- | --- | --- | --- | --- | --- | --- | --- | --- | --- |
|  | % | N | % | N | % | N | % | N | % | N |
| 0-5 | 38% | 5 | 0% | 0 | 38% | 5 | 23% | 3 | 0% | 0 |
| 6-10 | 0% | 0 | 13% | 2 | 53% | 8 | 13% | 2 | 20% | 3 |
| 11-15 | 5% | 1 | 20% | 4 | 25% | 5 | 15% | 3 | 35% | 7 |

**Table M in S2 Table**

**Table N in S2 Table**

| Years | Further Training | | Not Related to Science | | Primarily Research | | Primarily Teaching | | Science Related | |
| --- | --- | --- | --- | --- | --- | --- | --- | --- | --- | --- |
|  | % | N | % | N | % | N | % | N | % | N |
| 0-5 | 28% | 34 | 11% | 13 | 24% | 29 | 13% | 16 | 23% | 28 |
| 6-10 | 14% | 26 | 8% | 15 | 30% | 55 | 17% | 31 | 30% | 54 |
| 11-15 | 2% | 3 | 14% | 23 | 35% | 57 | 18% | 29 | 31% | 51 |

**Table O in S2 Table**

| Years | Further Training | | Not Related to Science | | Primarily Research | | Primarily Teaching | | Science Related | |
| --- | --- | --- | --- | --- | --- | --- | --- | --- | --- | --- |
|  | % | N | % | N | % | N | % | N | % | N |
| 0-5 | 29% | 35 | 7% | 9 | 27% | 33 | 16% | 19 | 21% | 25 |
| 6-10 | 11% | 20 | 9% | 17 | 30% | 54 | 19% | 35 | 31% | 56 |
| 11-15 | 1% | 2 | 14% | 24 | 33% | 58 | 18% | 32 | 34% | 59 |

**Table P in S2 Table**

| Years | Further Training | | Not Related to Science | | Primarily Research | | Primarily Teaching | | Science Related | |
| --- | --- | --- | --- | --- | --- | --- | --- | --- | --- | --- |
|  | % | N | % | N | % | N | % | N | % | N |
| 0-5 | 43% | 50 | 6% | 7 | 31% | 36 | 8% | 9 | 11% | 13 |
| 6-10 | 35% | 53 | 2% | 3 | 35% | 53 | 12% | 18 | 16% | 25 |
| 11-15 | 4% | 5 | 7% | 8 | 44% | 53 | 10% | 12 | 35% | 42 |

**Table Q in S2 Table**

| Years | Faculty Member (Tenure) | | Group Leader (Research) | | Health Care Provider | | Postdoctoral (Scientific Research) | | Research Staff or Technical Director | |
| --- | --- | --- | --- | --- | --- | --- | --- | --- | --- | --- |
|  | % | N | % | N | % | N | % | N | % | N |
| 0-5 | 12% | 29 | 3% | 7 | 7% | 16 | 36% | 85 | 17% | 40 |
| 6-10 | 23% | 76 | 5% | 17 | 13% | 44 | 22% | 74 | 13% | 42 |
| 11-15 | 33% | 96 | 11% | 31 | 13% | 38 | 3% | 9 | 11% | 32 |

**Table R in S2 Table**

| Years | Faculty Member (Tenure) | | Group Leader (Research) | | Health Care Provider | | Postdoctoral (Scientific Research) | | Research Staff or Technical Director | |
| --- | --- | --- | --- | --- | --- | --- | --- | --- | --- | --- |
|  | % | N | % | N | % | N | % | N | % | N |
| 0-5 | 11% | 11 | 5% | 5 | 1% | 1 | 46% | 46 | 19% | 19 |
| 6-10 | 24% | 38 | 4% | 7 | 9% | 14 | 25% | 39 | 15% | 23 |
| 11-15 | 36% | 53 | 13% | 19 | 10% | 15 | 3% | 4 | 12% | 18 |

**Table S**

| Years | Faculty Member (Tenure) | | Group Leader (Research) | | Health Care Provider | | Postdoctoral (Scientific Research) | | Research Staff or Technical Director | |
| --- | --- | --- | --- | --- | --- | --- | --- | --- | --- | --- |
|  | % | N | % | N | % | N | % | N | % | N |
| 0-5 | 13% | 18 | 1% | 2 | 11% | 15 | 28% | 39 | 15% | 21 |
| 6-10 | 22% | 38 | 6% | 10 | 17% | 30 | 20% | 35 | 11% | 19 |
| 11-15 | 29% | 43 | 8% | 12 | 16% | 23 | 3% | 5 | 10% | 14 |

**Table T in S2 Table**

| Years | Faculty Member (Tenure) | | Group Leader (Research) | | Health Care Provider | | Postdoctoral (Scientific Research) | | Research Staff or Technical Director | |
| --- | --- | --- | --- | --- | --- | --- | --- | --- | --- | --- |
|  | % | N | % | N | % | N | % | N | % | N |
| 0-5 | 8% | 8 | 3% | 3 | 3% | 3 | 44% | 43 | 21% | 20 |
| 6-10 | 18% | 24 | 4% | 5 | 8% | 10 | 36% | 47 | 16% | 21 |
| 11-15 | 31% | 33 | 13% | 14 | 11% | 12 | 4% | 4 | 11% | 12 |

**Table U in S2 Table**

| Years | Faculty Member (Tenure) | | Group Leader (Research) | | Health Care Provider | | Postdoctoral (Scientific Research) | | Research Staff or Technical Director | |
| --- | --- | --- | --- | --- | --- | --- | --- | --- | --- | --- |
|  | % | N | % | N | % | N | % | N | % | N |
| 0-5 | 7% | 1 | 15% | 2 | 8% | 1 | 38% | 5 | 23% | 3 |
| 6-10 | 8% | 1 | 7% | 1 | 13% | 2 | 0% | 0 | 27% | 4 |
| 11-15 | 30% | 6 | 10% | 2 | 15% | 3 | 10% | 2 | 0% | 0 |

**Table V in S2 Table**

| Years | Faculty Member (Tenure) | | Group Leader (Research) | | Health Care Provider | | Postdoctoral (Scientific Research) | | Research Staff or Technical Director | |
| --- | --- | --- | --- | --- | --- | --- | --- | --- | --- | --- |
|  | % | N | % | N | % | N | % | N | % | N |
| 0-5 | 17% | 20 | 2% | 2 | 10% | 12 | 28% | 34 | 12% | 14 |
| 6-10 | 28% | 51 | 6% | 11 | 17% | 31 | 14% | 26 | 9% | 17 |
| 11-15 | 35% | 57 | 8% | 13 | 12% | 20 | 2% | 3 | 11% | 18 |

**Table W in S2 Table**

| Years | Faculty Member (Tenure) | | Group Leader (Research) | | Health Care Provider | | Postdoctoral (Scientific Research) | | Research Staff or Technical Director | |
| --- | --- | --- | --- | --- | --- | --- | --- | --- | --- | --- |
|  | % | N | % | N | % | N | % | N | % | N |
| 0-5 | 17% | 20 | 2% | 2 | 12% | 14 | 29% | 35 | 14% | 17 |
| 6-10 | 25% | 46 | 6% | 11 | 18% | 32 | 11% | 20 | 10% | 18 |
| 11-15 | 34% | 60 | 7% | 13 | 15% | 27 | 2% | 3 | 10% | 18 |

**Table X in S2 Table**

| Years | Faculty Member (Tenure) | | Group Leader (Research) | | Health Care Provider | | Postdoctoral (Scientific Research) | | Research Staff or Technical Director | |
| --- | --- | --- | --- | --- | --- | --- | --- | --- | --- | --- |
|  | % | N | % | N | % | N | % | N | % | N |
| 0-5 | 8% | 9 | 4% | 5 | 2% | 2 | 43% | 50 | 20% | 23 |
| 6-10 | 20% | 30 | 4% | 6 | 8% | 12 | 36% | 54 | 16% | 24 |
| 11-15 | 30% | 36 | 15% | 18 | 9% | 11 | 5% | 6 | 12% | 14 |
