# Supplementary material for: Visualization of gender, race, citizenship and academic performance in association with career outcomes of 15-year biomedical doctoral alumni at a public research university
Source: PLoS One. 2018 May 17;13(5):e0197473. doi: 10.1371/journal.pone.0197473 (PMC5957427; doi:10.1371/journal.pone.0197473)
Supplement: S3 Table — Analyses are shown in Tables A-D for Employment Sector (Table A for total; Table B by gender; Table C by race, and Table D by U.S. citizenship status); Tables E-H for Career Type (Table E for total; Table F by gender; Table G by race, and Table H by U.S. citizenship status); and Tables I-L for Job Function (Table I for total; Table J by gender; Table K by race, and Table L by U.S. citizenship status). Neither GRE-Q nor GRE-V significantly predicted the likelihood of entering the Academic or For-Profit Employment Sector (GRE-Q B = -.004, SE = .018, Wald = .06, p = .81; GRE-V, B = -.016, SE = .016, Wald = 1.02, p = .31. Neither GPA nor TTD significantly predicted the likelihood of employment the Academic or For-Profit Employment Sector, (GPA B = -.016, SE = .342, Wald = .032, p = .86; TTD B = -.027, SE = .041, Wald = .444, p = .51). (DOCX) [file pone.0197473.s003.docx]

**S3 Tables**

**Table A in S3 Table**

| Employment Sector | | | | 1  (0-5 years) | | 2  (6-10 years) | | 3  (11-15 years) | | Total |
| --- | --- | --- | --- | --- | --- | --- | --- | --- | --- | --- |
|  | Academia | Count | 231^a^ | | 163^a, b^ | | 96^b^ | | 490 | |
|  |  | % | 64.0% | | 54.9% | | 46.4% | | 56.6% | |
|  | For-Profit | Count | 96^a^ | | 103^a, b^ | | 90^b^ | | 289 | |
|  |  | % | 26.6% | | 34.7% | | 43.5% | | 33.4% | |
|  | Government | Count | 21^a^ | | 15^a^ | | 11^a^ | | 47 | |
|  |  | % | 5.8% | | 5.1% | | 5.3% | | 5.4% | |
|  | Nonprofit | Count | 13^a^ | | 16^a^ | | 10^a^ | | 39 | |
|  |  | % | 3.6% | | 5.4% | | 4.8% | | 4.5% | |
| Total | | Count | | 361 | | 297 | | 207 | | 865 |
|  |  | % | | 100.0% | | 100.0% | | 100.0% | | 100.0% |

X^2^ (6, N = 865) = 20.30, p = .002. Cells within the same row that share different superscripts are significantly different from each other at the p < .05 level.

**Table B in S3 Table**

| Employment Sector | | | Female | Male | | Total |
| --- | --- | --- | --- | --- | --- | --- |
|  | Academia | Count | 264^a^ | 226^a^ | 490 | |
|  |  | % | 57.5% | 55.7% | 56.6% | |
|  | For-Profit | Count | 143^a^ | 146^a^ | 289 | |
|  |  | % | 31.2% | 36.0% | 33.4% | |
|  | Government | Count | 26^a^ | 21^a^ | 47 | |
|  |  | % | 5.7% | 5.2% | 5.4% | |
|  | Nonprofit | Count | 26^a^ | 13^a^ | 39 | |
|  |  | % | 5.7% | 3.2% | 4.5% | |
| Total | | Count | 459 | 406 | | 865 |
|  |  | % | 100.0% | 100.0% | | 100.0% |

X^2^ (3, N = 865) = 4.61, p = .202. Cells within the same row that share different superscripts are significantly different from each other at the p < .05 level.

**Table C in S3 Table**

| Employment Sector | | | Asian | Black or African American | White | Total |
| --- | --- | --- | --- | --- | --- | --- |
|  | Academia | Count | 207^a^ | 26^a, b^ | 247^b^ | 480 |
|  |  | % | 62.0% | 54.2% | 53.2% | 56.7% |
|  | For-Profit | Count | 92^a^ | 13^a, b^ | 177^b^ | 282 |
|  |  | % | 27.5% | 27.1% | 38.1% | 33.3% |
|  | Government | Count | 22^a, b^ | 7^b^ | 18^a^ | 47 |
|  |  | % | 6.6% | 14.6% | 3.9% | 5.6% |
|  | Nonprofit | Count | 13^a^ | 2^a^ | 22^a^ | 37 |
|  |  | % | 3.9% | 4.2% | 4.7% | 4.4% |
| Total | | Count | 334 | 48 | 464 | 846 |
|  |  | % | 100.0% | 100.0% | 100.0% | 100.0% |

X^2^ (6, N = 846) = 20.17, p = .003. Cells within the same row that share different superscripts are significantly different from each other at the p < .05 level.

**Table D in S3 Table**

| Employment Sector | | | US Citizens | Non-US Citizens | Total |
| --- | --- | --- | --- | --- | --- |
|  | Academia | Count | 262^a^ | 228^a^ | 490 |
|  |  | % | 54.8% | 58.9% | 56.6% |
|  | For-Profit | Count | 167^a^ | 122^a^ | 289 |
|  |  | % | 34.9% | 31.5% | 33.4% |
|  | Government | Count | 26^a^ | 21^a^ | 47 |
|  |  | % | 5.4% | 5.4% | 5.4% |
|  | Nonprofit | Count | 23^a^ | 16^a^ | 39 |
|  |  | % | 4.8% | 4.1% | 4.5% |
| Total | | Count | 478 | 387 | 865 |
|  |  | % | 100.0% | 100.0% | 100.0% |

X^2^ (3, N = 865) = 1.60, p = .660. Cells within the same row that share different superscripts are significantly different from each other at the p < .05 level.

**Table E in S3 Table**

| Career Type | | | 1  (0-5 years) | 2  (6-10 years) | 3  (11-15 years) | Total |
| --- | --- | --- | --- | --- | --- | --- |
|  | Further Training or Education | Count | 126^a^ | 34^b^ | 5^c^ | 165 |
|  |  | % | 34.9% | 11.4% | 2.4% | 19.1% |
|  | Not Related to Science | Count | 21^a^ | 22^a, b^ | 25^b^ | 68 |
|  |  | % | 5.8% | 7.4% | 12.1% | 7.9% |
|  | Primarily Research | Count | 105^a^ | 110^a^ | 72^a^ | 287 |
|  |  | % | 29.1% | 37.0% | 34.8% | 33.2% |
|  | Primarily Teaching | Count | 42^a^ | 52^a^ | 31^a^ | 125 |
|  |  | % | 11.6% | 17.5% | 15.0% | 14.5% |
|  | Science-Related | Count | 67^a^ | 79^b^ | 74^b^ | 220 |
|  |  | % | 18.6% | 26.6% | 35.7% | 25.4% |
| Total | | Count | 361 | 297 | 207 | 865 |
|  |  | % | 100.0% | 100.0% | 100.0% | 100.0% |

X^2^ (8, N = 865) = 116.05, p = .0001. Cells within the same row that share different superscripts are significantly different from each other at the p < .05 level.

**Table F in S3 Table**

| Career Type | | | Female | Male | Total |
| --- | --- | --- | --- | --- | --- |
|  | Further Training or Education | Count | 77^a^ | 88^a^ | 165 |
|  |  | % | 16.8% | 21.7% | 19.1% |
|  | Not Related to Science | Count | 42^a^ | 26^a^ | 68 |
|  |  | % | 9.2% | 6.4% | 7.9% |
|  | Primarily Research | Count | 135^a^ | 152^b^ | 287 |
|  |  | % | 29.4% | 37.4% | 33.2% |
|  | Primarily Teaching | Count | 87^a^ | 38^b^ | 125 |
|  |  | % | 19.0% | 9.4% | 14.5% |
|  | Science-Related | Count | 118^a^ | 102^a^ | 220 |
|  |  | % | 25.7% | 25.1% | 25.4% |
| Total | | Count | 459 | 406 | 865 |
|  |  | % | 100.0% | 100.0% | 100.0% |

X^2^ (3, N = 865) = 22.72, p = .0001. Cells within the same row that share different superscripts are significantly different from each other at the p < .05 level.

**Table G in S3 Table**

| Career Type | | | Asian | Black or African American | White | Total |
| --- | --- | --- | --- | --- | --- | --- |
|  | Further Training or Education | Count | 92^a^ | 6^a, b^ | 63^b^ | 161 |
|  |  | % | 27.5% | 12.5% | 13.6% | 19.0% |
|  | Not Related to Science | Count | 11^a^ | 6^b^ | 51^b^ | 68 |
|  |  | % | 3.3% | 12.5% | 11.0% | 8.0% |
|  | Primarily Research | Count | 122^a^ | 18^a^ | 141^a^ | 281 |
|  |  | % | 36.5% | 37.5% | 30.4% | 33.2% |
|  | Primarily Teaching | Count | 38^a^ | 8^a^ | 76^a^ | 122 |
|  |  | % | 11.4% | 16.7% | 16.4% | 14.4% |
|  | Science-Related | Count | 71^a^ | 10^a^ | 133^a^ | 214 |
|  |  | % | 21.3% | 20.8% | 28.7% | 25.3% |
| Total | | Count | 334 | 48 | 464 | 846 |
|  |  | % | 100.0% | 100.0% | 100.0% | 100.0% |

X^2^ (8, N = 846) = 47.27, p = .0001. Cells within the same row that share different superscripts are significantly different from each other at the p < .05 level.

**Table H in S3 Table**

| Career Type | | | US Citizens | Non-US Citizens | Total |
| --- | --- | --- | --- | --- | --- |
|  | Further Training or Education | Count | 57^a^ | 108^b^ | 165 |
|  |  | % | 11.9% | 27.9% | 19.1% |
|  | Not Related to Science | Count | 50^a^ | 18^b^ | 68 |
|  |  | % | 10.5% | 4.7% | 7.9% |
|  | Primarily Research | Count | 145^a^ | 142^b^ | 287 |
|  |  | % | 30.3% | 36.7% | 33.2% |
|  | Primarily Teaching | Count | 86^a^ | 39^b^ | 125 |
|  |  | % | 18.0% | 10.1% | 14.5% |
|  | Science-Related | Count | 140^a^ | 80^b^ | 220 |
|  |  | % | 29.3% | 20.7% | 25.4% |
| Total | | Count | 478 | 387 | 865 |
|  |  | % | 100.0% | 100.0% | 100.0% |

X^2^ (4, N = 865) = 55.94, p = .0001. Cells within the same row that share different superscripts are significantly different from each other at the p < .05 level.

**Table I in S3 Table**

| Job Functions | | | 1  (0-5 years) | 2  (6-10 years) | 3  (11-15 years) | Total |
| --- | --- | --- | --- | --- | --- | --- |
|  | Faculty Member - Tenure/Tenure Track | Count | 50^a^ | 85^b^ | 66^b^ | 201 |
|  |  | % | 18.1% | 38.6% | 47.5% | 31.6% |
|  | Group Leader (Research) | Count | 12^a^ | 26^b^ | 17^b^ | 55 |
|  |  | % | 4.3% | 11.8% | 12.2% | 8.6% |
|  | Healthcare Provider | Count | 34^a^ | 35^a^ | 29^a^ | 98 |
|  |  | % | 12.3% | 15.9% | 20.9% | 15.4% |
|  | Postdoctoral (Scientific Research) | Count | 127^a^ | 34^b^ | 7^c^ | 168 |
|  |  | % | 45.8% | 15.5% | 5.0% | 26.4% |
|  | Research Staff or Technical Director | Count | 54^a^ | 40^a^ | 20^a^ | 114 |
|  |  | % | 19.5% | 18.2% | 14.4% | 17.9% |
| Total | | Count | 277 | 220 | 139 | 636 |
|  |  | % | 100.0% | 100.0% | 100.0% | 100.0% |

X^2^ (8, N = 636) = 120.72, p = .0001. Cells within the same row that share different superscripts are significantly different from each other at the p < .05 level.

**Table J in S3 Table**

| Job Functions | | | Female | Male | Total |
| --- | --- | --- | --- | --- | --- |
|  | Faculty Member - Tenure/Tenure Track | Count | 99^a^ | 102^a^ | 201 |
|  |  | % | 30.6% | 32.7% | 31.6% |
|  | Group Leader (Research) | Count | 24^a^ | 31^a^ | 55 |
|  |  | % | 7.4% | 9.9% | 8.6% |
|  | Healthcare Provider | Count | 68^a^ | 30_b_ | 98 |
|  |  | % | 21.0% | 9.6% | 15.4% |
|  | Postdoctoral (Scientific Research) | Count | 79^a^ | 89^a^ | 168 |
|  |  | % | 24.4% | 28.5% | 26.4% |
|  | Research Staff or Technical Director | Count | 54^a^ | 60^a^ | 114 |
|  |  | % | 16.7% | 19.2% | 17.9% |
| Total | | Count | 324 | 312 | 636 |
|  |  | % | 100.0% | 100.0% | 100.0% |

X^2^ (4, N = 636) = 16.36, p = .003. Cells within the same row that share different superscripts are significantly different from each other at the p < .05 level.

**Table K in S3 Table**

| Job Functions | | | Asian | Black or African American | White | Total |
| --- | --- | --- | --- | --- | --- | --- |
|  | Faculty Member - Tenure/Tenure Track | Count | 65^a^ | 8^a, b^ | 128^b^ | 201 |
|  |  | % | 25.1% | 24.2% | 38.9% | 32.4% |
|  | Group Leader (Research) | Count | 22^a^ | 5^a^ | 26^a^ | 53 |
|  |  | % | 8.5% | 15.2% | 7.9% | 8.5% |
|  | Healthcare Provider | Count | 25^a^ | 6^a, b^ | 63^b^ | 94 |
|  |  | % | 9.7% | 18.2% | 19.1% | 15.1% |
|  | Postdoctoral (Scientific Research) | Count | 94^a^ | 7^a, b^ | 63^b^ | 164 |
|  |  | % | 36.3% | 21.2% | 19.1% | 26.4% |
|  | Research Staff or Technical Director | Count | 53^a^ | 7^a^ | 49^a^ | 109 |
|  |  | % | 20.5% | 21.2% | 14.9% | 17.6% |
| Total | | Count | 259 | 33 | 329 | 621 |
|  |  | % | 100.0% | 100.0% | 100.0% | 100.0% |

X^2^ (8, N = 621) = 39.26, p = .0001. Cells within the same row that share different superscripts are significantly different from each other at the p < .05 level.

**Table L** **in S3 Table**

| Job Functions | | | US Citizens | Non-US Citizens | Total |
| --- | --- | --- | --- | --- | --- |
|  | Faculty Member - Tenure/Tenure Track | Count | 126^a^ | 75^b^ | 201 |
|  |  | % | 37.5% | 25.0% | 31.6% |
|  | Group Leader (Research) | Count | 26^a^ | 29^a^ | 55 |
|  |  | % | 7.7% | 9.7% | 8.6% |
|  | Healthcare Provider | Count | 73^a^ | 25^b^ | 98 |
|  |  | % | 21.7% | 8.3% | 15.4% |
|  | Postdoctoral (Scientific Research) | Count | 58^a^ | 110^b^ | 168 |
|  |  | % | 17.3% | 36.7% | 26.4% |
|  | Research Staff or Technical Director | Count | 53^a^ | 61^a^ | 114 |
|  |  | % | 15.8% | 20.3% | 17.9% |
| Total | | Count | 336 | 300 | 636 |
|  |  | % | 100.0% | 100.0% | 100.0% |

X^2^ (4, N = 865) = 51.40, p = .0001. Cells within the same row that share different superscripts are significantly different from each other at the p < .05 level.
