# Supplementary material for: Visualization of gender, race, citizenship and academic performance in association with career outcomes of 15-year biomedical doctoral alumni at a public research university
Source: PLoS One. 2018 May 17;13(5):e0197473. doi: 10.1371/journal.pone.0197473 (PMC5957427; doi:10.1371/journal.pone.0197473)
Supplement: S1 File — (PDF) [file pone.0197473.s004.pdf]

S1 File.

Census_2015

Start of Block: Demographic Block

Q1 **Wayne State University Graduate School Alumni Census**

Q2 Demographic information on File:

Last Name: **${m://LastName}**
First Name: **${m://FirstName}**
ID: **${e://Field/ID}**

If your name has changed since you graduated, your name while you were at WSU:
${e://Field/LastName}

preferred email address: **${m://Email1}**

Current Address: **missing**

Q3 Graduate Degree Information (if you received more than one WSU degree, the most recent degree will be listed):

Department/Program: **${e://Field/DEPARTMENTDESC}**
Degree Awarded: **${e://Field/DEGREE}**
Degree Date: **${e://Field/ACADEMIC_PERIOD_GRADUATION}**

Q4 Please update the following parts of my contact information: (you will be prompted to provide information for any field you indicate)

- ▢ name (1)
- ▢ email address (2)
- ▢ postal address (3)
- ▢ department/degree information (6)
- ▢ nothing to change (4)

Q5 I use LinkedIN to maintain my professional social network?

- ○ Yes (1)
- ○ No (2)

Q6 I wish to stay connected with WSU through LinkedIN. My profile name is...

____

Q7 I do not wish to be contacted by WSU in the future (opt out from future mailings or surveys).

- ○ True (1)
- ○ False (2)

Page Break

Display This Question:

If Please update the following parts of my contact information: (you will be prompted to provide inf... = name

Q8 Last Name:

____

Display This Question:

If Please update the following parts of my contact information: (you will be prompted to provide inf... = name

Q9 First Name:

____

Display This Question:

If Please update the following parts of my contact information: (you will be prompted to provide inf... = name

Q10 Middle Name:

____

Display This Question:

If Please update the following parts of my contact information: (you will be prompted to provide inf... = email address

*

Q11 Preferred email address:

____

Page Break

Display This Question:

If Please update the following parts of my contact information: (you will be prompted to provide inf... = postal address

Q12 Address Line 1:

____

Display This Question:

If Please update the following parts of my contact information: (you will be prompted to provide inf... = postal address

Q13 Address Line 2:

____

Display This Question:

If Please update the following parts of my contact information: (you will be prompted to provide inf... = postal address

Q14 Address Line 3:

____

Display This Question:

If Please update the following parts of my contact information: (you will be prompted to provide inf... = postal address

Q15 City:

____

Display This Question:

If Please update the following parts of my contact information: (you will be prompted to provide inf... = postal address

Q16 State:

____

Display This Question:

If Please update the following parts of my contact information: (you will be prompted to provide inf... = postal address

Q17 Zipcode:

____

Display This Question:

If Please update the following parts of my contact information: (you will be prompted to provide inf... = postal address

Q18 Country:

____

Display This Question:

If Please update the following parts of my contact information: (you will be prompted to provide inf... = department/degree information

Q19 Please indicate what is in error about the degree information listed.

____

Page Break

End of Block: Demographic Block

Start of Block: Short Form

Q20 **Short Form Census:**

Q21 Current data WSU has on file regarding your employment:

Current Employer: **${e://Field/currentemployer}**

At current employer since: **MISSING**

Current Job Title: **${e://Field/currentjobtitle}**

In current job title since: **MISSING**

Field in which you work: **MISSING**

Geographic local: **MISSING**

Q22 Is all of the information listed above correct and current?

- ◯ Yes (1)
- ◯ No (2)

Q23 Which of the following categories best describes your current employment status?

▼ - (1) ... Other (9)

Q24 Which statement best categorizes the terms of your employment?

▼ - (1) ... Currently employed but seeking employment (8)

Display This Question:

If Is all of the information listed above correct and current? = No

Q25 Current employer

____

Q26 In what year where you hired by current employer?

▼ - (1) ... 2015 (22)

Q27 Current job title:

____

Q28 In what year did you assume your current job title?

▼ 1995 (1) ... 2015 (21)

Q29 Which answer best describes the geographical location of employer

▼ Detroit (1) ... other (12)

Q30 Employer category:

▼ - (27) ... other (26)

Q31 If you listed the employer category as other, please elaborate.

____

Page Break

End of Block: Short Form

Start of Block: Long Form

Q32 **Additional Questions for Long Form Census:**

Q33 You have been randomly selected to complete the long-form of the PhD alumni census. If you are willing, the additional questions will take about 10 minutes to complete. The longer version asks more detailed questions about how your training at Wayne State prepared you for your first position after graduate school as well as your current position. We will use these data to help us improve our doctoral programs.

Are you willing to complete the longer questionnaire?

- ○ Yes - I agree to complete the long version (1)
- ○ No - I would rather stop here (2)

Skip To: End of Block If You have been randomly selected to complete the long-form of the PhD alumni census. If you are wi... = No - I would rather stop here

Page Break

Q34 Were you unemployed and looking for work at any time during 2014?

- Yes (1)
- No (2)

Q35 Which category best describes your residency status while at Wayne State University?

- Native U.S. Citizen (1)
- Naturalized U.S. Citizen (2)
- Canadian Citizen (3)
- permanent resident/green card holder (4)
- Student Visa (5)
- Spousal Visa (6)
- Other visa type (7)

Q36 Which category best describes your current residency status?

- ○ Native U.S. Citizen (1)
- ○ Naturalized U.S. Citizen (2)
- ○ Canadian Citizen (3)
- ○ Permanent resident/green card holder (4)
- ○ Student Visa (5)
- ○ Spousal Visa (6)
- ○ Other visa type (7)
- ○ Living or working outside of the U.S. (8)

Page Break

Q38 I have held ___ employment positions since leaving Wayne State.

▼ 0 (1) ... 10+ (8)

Q39 Immediately after graduate school I…

▼ - (1) ... other (please specify) (11)

Q40 Please specify:

____

Q41 I held my first position after WSU for ___ year(s).

▼ 0 (1) ... 10+ (6)

Q42 I have been in my current position for ___ year(s).

▼ 0 (1) ... 10+ (6)

Page Break

Q37 On a scale of 1 to 5, rate whether you agree or disagree with the following statements:

	Strongly Agree (1)	Agree (2)	Neither Agree nor Disagree (3)	Disagree (4)	Strongly Disagree (5)
I enjoyed my time in graduate school at Wayne State. (1)	○	○	○	○	○
My Ph.D. advisor respected me as a student and a colleague. (2)	○	○	○	○	○
My Ph.D. advisor acted in my best interests to help me develop in my field. (3)	○	○	○	○	○
The Ph.D. program requirements were clearly explained to me. (4)	○	○	○	○	○
The courses I took at WSU provided me with background necessary to perform my research. (5)	○	○	○	○	○
The courses I took at WSU provided me with material needed to pass my qualifying exam. (6)	○	○	○	○	○
My experience at WSU prepared me well for my first position after graduate school. (7)	○	○	○	○	○
My training at WSU prepared me well for my current position. (8)	○	○	○	○	○
I would recommend my program at WSU to a friend, colleague or advisee. (9)	○	○	○	○	○

Relative to new graduates I met from other Ph.D. programs, my presentation skills were better than theirs, thanks to my experience at WSU. (10)	○	○	○	○	○
Relative to new graduates I met from other Ph.D. programs, my writing skills were better than theirs, thanks to my experience at WSU. (11)	○	○	○	○	○
Relative to new graduates I met from other Ph.D. programs, my disciplinary skills were better than theirs, thanks to my experience at WSU. (12)	○	○	○	○	○
Relative to new graduates I met from other Ph.D. programs, my critical thinking skills were better than theirs, thanks to my experience at WSU. (13)	○	○	○	○	○
The facilities at WSU were better than those I have had the opportunity to use in my post-Wayne positions. (14)	○	○	○	○	○
Were I in a position to hire a Ph.D., I would look favorably on a WSU Ph.D. recipient for that position. (15)	○	○	○	○	○
People in my company/university/place of employment who are in a position to hire new Ph.D. recipients look favorably on WSU alumni such as myself. (16)	○	○	○	○	○

If I had the opportunity to do it over again, I would still have pursued a Ph.D. in my chosen field. (17)	○	○	○	○	○
If I had the opportunity to do it over again, I would have still have gotten my Ph.D. from Wayne State? (18)	○	○	○	○	○

End of Block: Long Form

Start of Block: Submit form

Q43 Please share with the Graduate School anything about your significant achievements or accomplishments since Wayne State?

____

____

____

____

____

Q44 The Graduate School is interested in using the alumni network to help mentor new doctoral students through graduate professional development activities. Would you be interested in interacting with current doctoral students or recent alumni through career panels or other related activities?

○ Yes (1)

○ No (2)

Q45 Do you have any feedback that you would like shared anonymously with the faculty of your graduate program or the graduate school in general?

____

____

____

____

____

Q46 Submit Survey?

- ○ Yes (1)
- ○ No (2)

End of Block: Submit form
